# Advances in Non-Viral DNA Vectors for Gene Therapy

**DOI:** 10.3390/genes8020065

**Published:** 2017-02-10

**Authors:** Cinnamon L. Hardee, Lirio Milenka Arévalo-Soliz, Benjamin D. Hornstein, Lynn Zechiedrich

**Affiliations:** 1 Interdepartmental Program in Integrative Molecular and Biomedical Sciences, Baylor College of Medicine, Houston, TX 77030, USA; Cinnamon.Hardee@bcm.edu; 2 Department of Molecular Virology and Microbiology, Baylor College of Medicine, Houston, TX 77030, USA; lmareval@bcm.edu (L.M.A.-S.); hornstei@bcm.edu (B.D.H.); 3 Verna and Marrs McLean Department of Biochemistry and Molecular Biology, Baylor College of Medicine, Houston, TX 77030, USA; 4 Department of Pharmacology, Baylor College of Medicine, Houston, TX 77030, USA

**Keywords:** minimized vector, antibiotic-free plasmid, miniplasmid, minicircle, minivector, DNA vaccine

## Abstract

Uses of viral vectors have thus far eclipsed uses of non-viral vectors for gene therapy delivery in the clinic. Viral vectors, however, have certain issues involving genome integration, the inability to be delivered repeatedly, and possible host rejection. Fortunately, development of non-viral DNA vectors has progressed steadily, especially in plasmid vector length reduction, now allowing these tools to fill in specifically where viral or other non-viral vectors may not be the best options. In this review, we examine the improvements made to non-viral DNA gene therapy vectors, highlight opportunities for their further development, address therapeutic needs for which their use is the logical choice, and discuss their future expansion into the clinic.

## 1. Introduction to Gene Therapy

Gene therapy is the use of nucleic acids to repair, replace, or regulate genes to prevent or treat disease [1]. Hundreds of genes have been investigated as potential gene therapy candidates. Some notable examples include replacement of the mutated cystic fibrosis transmembrane conductance regulator with a functional copy to treat cystic fibrosis [2], the knockdown of C–C chemokine receptor type 5 (CCR5) to block cell entry by human immunodeficiency virus (HIV) [3], the expression of glucagon-like peptide 1 mimetics to regulate blood glucose levels in diabetic patients [4], and expression of viral antigens in a DNA vaccine for influenza that eliminates the need for potentially active viral particles [5].

Gene therapy vectors are broadly categorized as viral or non-viral [1]. Viral vectors are widely used because of their natural ability to invade cells and deliver a manipulated genetic payload for therapeutic use. It is far more difficult for non-viral vectors (RNA or DNA) to transfect many specific cell types, so they are usually complexed with delivery vehicles (e.g., cationic lipids, cationic polymers, etc.) or subjected to forced entry (e.g., electroporation, hydrodynamic injection, etc.). Advancements in transfection methods are occurring rapidly; for an overview of these delivery technologies, we refer the reader to the following reviews [6,7,8,9].

Lentiviral and retroviral vectors, which are designed for insertion into the genome, bring a high risk of gene disruption [10,11]. Adeno-associated virus (AAV) vectors have a lower but still existent risk of insertional mutagenesis [12]. Adenoviral vectors are maintained episomally, which is advantageous [13], but can cause toxicity and immunogenicity [14]. Certain viral vectors, such as those specifically based on adenovirus serotype 5 (AdV5) or adeno-associated virus type 2 (AAV2), cannot be used because the virus is so widespread that many people have a pre-existing immunity [15]. In all of these families of viral vectors, even those with low seroprevalence in the human population, the same serotype of construct cannot be delivered repeatedly to the same patient because they will have developed an immunity to it [12,16,17].

Non-viral vectors are far less immunogenic than viral vectors [18]. RNA delivery for RNA interference (RNAi)—short interfering RNAs (siRNA), microRNA (miRNA), etc.—, however, comes with the difficulty of expense and scale because they turn over so quickly. Longer RNAs, such as synthetic messenger RNAs (mRNAs), which are delivered to the cell and translated in vivo, offer potential for expressing proteins. The nucleotides in these mRNA molecules must be modified to avoid immune detection through pattern recognition receptors such as Toll-like receptor 3 (TLR-3), TLR-7, TLR-8, and retinoic acid-inducible gene I (RIG-I) [19]. RNA vectors are typically less stable and more transient than DNA and thus require additional protecting measures (end-blocking, base modification, vehicle choice, etc.) [20]. These protection measures, however, may introduce their own confounding issues.

The enormous potential for plasmids as non-viral vectors for gene therapy has been recognized since at least 1990 [21]. Compared to viral and RNA-based vectors, plasmids are easier and cheaper to produce, ship, and store, and have a much longer shelf life. In fact, making viral constructs involves creation and utilization of plasmid intermediates for the formation of viral particles. The modular nature of plasmids also allows for straightforward molecular cloning, making them easy to manipulate and design for therapeutic use. Plasmids integrate at a rate of less than 10^−5^ stable integrants per transfected cell [22] and, unlike viruses, can be delivered repeatedly. The important advantages of non-viral DNA vectors over viral vectors and RNA-based vectors have compelled researchers to work to improve their safety and utility. Because of improved safety over viral vectors, plasmids have enabled a number of clinical trails (Table 1). The goal of this review is to highlight improvements to non-viral DNA vectors, outline specific clinical situations best served by these vectors, and point out their possible future optimization and expansion into the clinic.

## 2. Challenges of Using Plasmid Vectors for Gene Therapy

Plasmid vectors for gene therapy are beset with some notable inherent limitations. Most plasmid DNA preparations contain several topological variants of the plasmid, including supercoiled (the preferred topology), but also the unwanted open circular and linear forms of the molecule. As mentioned above, plasmids are generally inefficient at delivering their payloads compared to viruses, thus requiring vehicles, physical forces, or specialized modifications for uptake and nuclear localization [25,26]. Some of these delivery methods lead to breakage of plasmid DNA backbone, which increases the likelihood of genome integration and, if the break occurs in the therapeutic sequence, less efficient expression [27].

Because plasmids are non-replicating episomes, transgene expression is transient and diluted by cell division. Additionally, bacterial sequences in plasmids can contribute to their gene silencing [28,29]. Unmethylated cytosine-phosphate-guanine (CpG) dinucleotides, which are more common in bacterial DNA than in mammalian DNA, have the potential to be recognized by the mammalian immune system via TLR-9, potentially precipitating not only transgene silencing but also immune response [30].

Plasmids contain a bacterial origin of replication (*ori*) for propagation in a bacterial host strain. An *ori* potentially allows plasmids meant to deliver therapeutic sequences to also inadvertently transfer into and replicate in other bacteria. Additionally, plasmids encode genes (typically antibiotic resistance-encoding genes) for selection of plasmid-harboring bacteria. The use of antibiotics and their resistance genes in the preparation of plasmid vectors, however, is discouraged by regulatory bodies such as the Food and Drug Administration and the European Medicines Agency because of the risk of transfer and replication of resistance genes to bacteria in the human microbiome and possibly into the environment. Additionally, residual antibiotics that remain from vector production may trigger an immune reaction in patients.

Because of the challenges, extensive modifications have been made to plasmids to satisfy regulatory requirements for clinical use in humans [18]. These modifications involved the deletion of unwanted and unnecessary sequences and have resulted in the advent of minimized DNA vectors.

## 3. Improvements to Plasmid Vectors

Several studies have revealed that decreasing plasmid size improves transfection efficiency, suggesting that minimizing vector length should be one of the goals of non-viral vector design [31,32,33]. Attempts to minimize and otherwise optimize plasmid DNA vectors for gene therapy first involved the removal of antibiotic resistance genes [34,35,36], thereby increasing clinical potential. Because of this removal, however, antibiotic-free systems had to utilize a different mechanism for selection. One of the first systems was operator repressor titration (ORT) [34]. ORT plasmids (pORT) contain one or more operator sequences that are used to titrate, through competition, repressor proteins (e.g., Lac repressor) that normally bind an endogenous operator sequence upstream of an essential, chromosomally encoded gene in bacteria.

In the case of pORT, the essential gene is *dapD*. Repression of *dapD* is lethal when cells are grown on specialized growth medium. Bacteria that harbor multi-copy plasmids outcompete the repressor from the endogenous operator and survive. This selectable marker system has only minimal bacterial sequences encoded on the plasmid (*ori* and short operator sequences), requires no plasmid gene expression, promotes plasmid stability, and, if need be for production, can be used in any microorganism able to propagate plasmids. pORT has been used effectively in pre-clinical and clinical studies as a DNA vaccine [37,38,39] and as a component of live bacterial vaccines using *Salmonella enterica* [40,41,42].

Other DNA vector selection systems that are free of antibiotic resistance genes rely on chromosomal mutations in special producer strains that are complemented only when plasmid is present. One of these systems is the plasmid with conditional origin of replication (pCOR) and another is the plasmid free of antibiotic resistance (pFAR). Both rely on amber mutations in essential chromosomal genes (the genes encoding arginine and thymidine, respectively), creating auxotrophic bacteria that only grow if they harbor plasmid containing the complementary amber suppressor tRNA.

The conditional origin of replication in pCOR, *ori*-γ from the R6K class A theta plasmid, relies upon a π initiator protein that is produced only within a narrow host range by the *pir* gene [43]. This safeguard means that pCOR would be unlikely to disseminate into the environment. Additionally, pCOR may be less immunostimulatory than standard ColE1-derived plasmids [35]. pCOR has demonstrated higher levels of reporter gene activity compared to commercially available plasmids [35] and also has been brought to clinical trial for critical limb ischemia [44]. Similarly, the pFAR system has been used to effect high luciferase expression in both transplanted tumor cells and in the skin of mice [36]. In both cases, pFAR vectors displayed higher gene expression and persistence than standard plasmid vectors.

By decreasing vector length, removal of antibiotic resistance sequences had the additional advantage of improving the efficiency of vector production in many cases because of decreased metabolic burden upon the bacterial host. Removal of these genes also meant that costly and time-consuming procedures for evaluating the amount of residual antibiotics left in vector preparations could be eliminated.

The vectors specifically mentioned above serve as highlighted examples; they and other antibiotic-free miniplasmid systems have been more thoroughly reviewed elsewhere [18,45,46]. In Table 2 we list DNA vectors and their general composition (whether they encode a bacterial origin of replication or an antibiotic resistance-encoding gene). Although we had hoped to include other key features (e.g., minimal and maximal vector length, expression efficiency, etc.) for all of the vectors, data were not consistently available. Therefore, we provide several distinguishing advantages and disadvantages of each.

## 4. Development of Minicircles and Minivectors

### 4.1. Minicircles

Despite the removal of some problematic sequences, significant bacterial sequences remain in antibiotic resistance-free systems. As mentioned above, bacterial sequences, and particularly *ori*, have the potential for triggering inflammation or for silencing transgenes [91]. Removal of these additional extraneous sequences was first reported in 1997 by Darquet et al. [65]. These researchers used site-specific recombination to turn parent plasmids (containing the regulatory sequences necessary for intramolecular recombination) into minicircles, containing the therapeutic sequences desired and only very short segments of bacterial DNA. The rest of the bacterial DNA from the parent plasmid, including *ori*, is recombined into a discarded miniplasmid. Because minicircles no longer contain *ori*, they cannot replicate in bacteria and are, thus, no longer considered plasmids.

Multiple recombinase systems have been used to generate minicircles, including phage λ integrase, phiC31 recombinase, Flp recombinase, ParA resolvase, and Cre recombinase (reviewed in [9,18]). In the miniplasmid systems mentioned in Section 3, the miniplasmids are, themselves, the gene therapy vector (because only antibiotic sequences were removed from them), whereas here they are discarded because they contain everything except the sequence of interest. In both cases, miniplasmids encode *ori*, and are therefore still referred to as plasmids.

Plasmid contaminants in minicircle preparations can be as high as 10% of the total yield—well above the 1.5% allowed by some health regulatory agencies [92]. Because of this problem, several methods for improving minicircle purity have been developed: (i) a triple helix DNA technology (TriD) that uses biotinylated oligonucleotides and streptavidin-coated magnetic beads to selectively remove parent plasmid and miniplasmid [92]; (ii) incorporation of unique nicking endonuclease sites outside of the minicircle sequence on the parent plasmid to facilitate separation of minicircles from contaminating products with hydrophobic interaction chromatography [93]; (iii) the use of an anion-exchange monolithic column (CIM diethylamine) for selective separation of minicircle from the unwanted products [94]; and (iv) enhancing ParA resolvase activity to increase yield during fermentation by boosting recombination for minicircle generation [95]. These methods all work to improve yield of minicircles, but add both time and expense to the preparation. Additional optimization may be needed to generate minicircles on an industrial scale.

### 4.2. Minivectors

**Figure 1:**
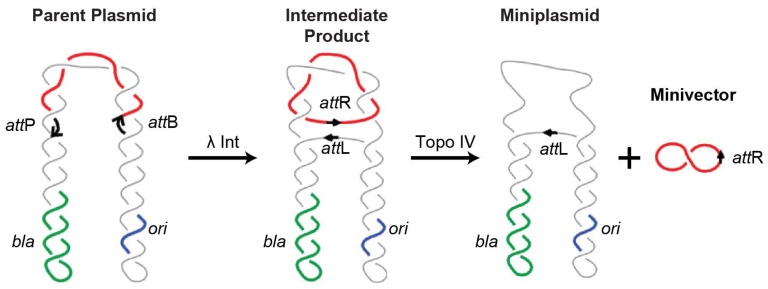
Generation of DNA minivectors. To generate minivectors, any target sequence or expression cassette is cloned between the *att*B and *att*P sites located in direct orientation in a minivector-producing parent plasmid. *λ* integrase mediates the intramolecular recombination of the *att*B and *att*P sites, producing two catenated rings: the minivector containing the target sequence or expression cassette, and a miniplasmid containing all the other undesired sequences. The catenanes are unlinked by topoisomerase IV. *λ* Int: lambda integrase; Topo IV: topoisomerase IV; *bla*: *β* lactamase (encoding ampicillin resistance); *ori*: bacterial origin of replication; *att*P: phage attachment site; *att*B: bacterial attachment site; *att*L: hybrid attachment site to the "left" of the recombined sequence; *att*R: hybrid attachment site to the "right" of the recombined sequence.

Minivectors are minimized, non-viral DNA vectors similar to minicircles but with some important differences. Like minicircles, minivectors are synthesized from a parent plasmid via site-specific recombination (Figure 1) [73]. Encoding only the genetic payload and short integration sequences, minivectors can be engineered as small as ~350 bp and generated in high yields (in comparison, the smallest reported minicircle length is 650 bp [32]; the yield of minicircles this small is unclear). As before, unwanted bacterial sequences are on a discarded miniplasmid. The recombination and purification system used to make minivectors is highly optimized, resulting in as much as 100-fold lower plasmid contamination than is recommended by health regulatory agencies [92].

### 4.3. Increasing the Functionality of Minimized DNA Vectors

Compared to plasmids, minicircles and minivectors transfect cells better in vitro, ex vivo, and in vivo [31,32,33], express transgenes more effectively [67,96], and have an improved safety profile for clinical use. In addition to having no problematic antibiotic resistance genes and negligible bacterial sequences, the benefits of reducing vector length for gene delivery and transgene expression have been well established [31,32,33,97]. Naked DNA vectors less than ~1,200 bp completely survive aerosolization, a direct consequence of being beneath a sharp threshold hydrodynamic radius [71]. Increased negative supercoiling makes minivectors even more compact, which both promotes nuclear localization and provides additional protection from the shear forces of aerosolization (in addition to the protection afforded by their smaller sizes) [71,98]. There is a dramatic length dependence for naked DNA vectors surviving human serum; the smaller the vector, the longer the survival [72,99]. For these reasons, then, minicircles and minivectors are a good choice for gene therapy trials. Additional modifications that could further improve minimized vectors follow.

#### 4.3.1. Nuclear Localization Signals

For a therapeutic vector to be efficiently delivered, it must first be able to reach the target cells. After that, a number of physical barriers must be traversed—the cell membrane, the cytoplasm, and, finally, the nuclear membrane. This last step is considered the rate-limiting step in transfection because most exogenous naked DNA (purified plasmids with no delivery vehicles) or complexed DNA vectors are too large to passively diffuse through the nuclear membrane [18,100].

Associating nuclear localization signals (NLSs) with non-viral DNA constructs would be useful for therapeutic applications, particularly in non-dividing cells. Found on proteins destined for the nucleus, NLSs are clusters of amino acid “marks” of positively charged residues, like arginine and lysine, that are recognized by karyopherins such as importin α. Karyopherins facilitate transport across the nuclear envelope through nuclear pore complexes. Plasmids mixed with cytoplasmic shuttle proteins containing NLSs are shuttled across the nuclear membrane, although at a rate at least two orders of magnitude slower than that of NLS-containing proteins alone [100]. This slow rate is probably a consequence of the time it takes for exogenous DNA and NLS-containing proteins to associate with each other inside the cell. If NLSs are added directly to DNA vectors, time into the nucleus could be reduced and gene expression increased [100].

There are several ways in which an NLS can be joined to a DNA construct [101,102]; some methods include electrostatic attraction, covalent linkage via direct chemical conjugation, or complexation of DNA and NLS-containing proteins using various linker molecules. In the latter strategy, Vaysse et al. took advantage of the high affinity of the tetracycline repressor (TetR) for the tetracycline operator sequence (*tetO*) [103]. They engineered fusions of the tetracycline repressor to both the well-studied SV40 large T antigen-derived NLS (TetR-NLS) and the HIV Tat (TetR-Tat) peptide. Upon sequential intravenous injection [104] of *lacZ*-expressing minicircles that also contained multiple *tetO* sequences, this group found a six-fold increase of β-galactosidase expression in murine lungs when minicircles were combined with the TetR-Tat fusion compared to parent plasmid injection alone [103]. in vitro, the transfection efficiency was more than 30-fold higher in A549 cells using minicircle and TetR-NLS compared to plasmid transfection alone [103]. The advances afforded by these proof-of-principle experiments will hopefully translate into the clinic in the future.

#### 4.3.2. Cytosine-Phosphate-Guanine Dinucleotides

A CpG motif contains a centralized cytosine-phosphate-guanine dinucleotide flanked by regions of different length and sequence depending on the type of CpG [105]. Unmethylated CpG motifs from bacterial DNA are pathogen-associated molecular patterns (PAMPs) that stimulate the innate immune response by triggering cells that contain TLR-9, such as human B-cells and plasmacytoid dendritic cells [106]. Members of the TLR family of proteins serve as pattern recognition receptors capable of detecting certain PAMPs. TLR-9, in particular, can detect unmethylated CpG motifs, which are at least four times more common in prokaryotic DNA than in eukaryotic DNA [107]. Thus, many plasmid vectors already contain an inbuilt source of immunogenicity that can be valuable for DNA vaccination.

CpG motifs have been encoded within plasmid DNA vaccines [108,109], or co-delivered with DNA vaccines on either other DNA vectors [110] or oligonucleotides (reviewed in [111,112]). Miniplasmid vectors have demonstrated utility as improved DNA vaccines [37,38,39]. Although minicircles have only been tested pre-clinically in a limited number of tests [113], interest in this application is mounting [27,70]. Minivectors have not yet been examined in this capacity to date. However, as with plasmid vectors above, CpG sequences can be added to minicircles or minivectors or delivered via additional oligonucleotides to promote an immunostimulatory effect when they are delivered as vaccines or co-delivered as adjuvants with other vaccines.

#### 4.3.3. Scaffold/Matrix Attachment Regions

Because minicircles and minivectors exist episomally and do not replicate on their own, their effectiveness dilutes with cell division or cell death. Although this transience is ideal for many therapeutic applications, others require more persistence. One way to circumvent the transient nature of minicircles and minivectors, particularly in quickly dividing cells (e.g., the hematopoietic system), would be to engineer them to contain scaffold/matrix attachment regions (S/MARs) [114,115]. S/MARs are endogenous AT-rich sequences that play an important role in the spatial organization of chromosomes through DNA loop base attachment to the nuclear matrix. S/MARs are often found close to regulatory elements such as promoters, enhancers, and origins of DNA replication [116]. S/MARs can be incorporated into DNA vectors to facilitate a once-per-cell-cycle replication to maintain the vector as an episome in daughter cells [116,117]. To confer function as an episome, an S/MAR sequence must be encoded downstream of an actively transcribed gene [117].

S/MAR sequences are ubiquitous and widely spread in eukaryotic genomes, with 453 in just one 30 Mb region of the human genome [118]. Because they are typically long sequences, averaging ~5 kb in length, their addition into minimized non-viral vectors would cause the loss of the advantages of small size. Furthermore, only a few S/MAR sequences have been validated to confer episomal vector maintenance. One such validated sequence, a ~2 kb S/MAR from the human β-interferon gene cluster, was successfully used to make a non-integrating lentiviral vector persist in dividing cells [119,120,121,122].

A direct comparison of a S/MAR-containing minicircle to a S/MAR-containing plasmid revealed that 65% of cells stably produced a green fluorescent protein (GFP) reporter 55 days post-transfection with the minicircle compared to only 3% of GFP-positive cells after transfection with the plasmid [123]. Cells transfected with minicircles without S/MAR lost luciferase expression in glioma cells after one week, but S/MAR-containing minicircles maintained expression for two months [117]. Similar results were seen in hydrodynamically injected mouse liver. In a direct comparison of minicircles and plasmids with and without S/MARs, only minicircles containing S/MAR expressed luciferase for 92 days [117]. Despite the length an S/MAR would add to an otherwise small vector, these successes illustrate the potential of S/MAR sequences to confer persistence for long-term gene therapy effects with vectors that otherwise do not replicate.

#### 4.3.4. Viral Replication Genes

The inclusion of viral sequences in episomal vector systems has granted them extended replication in mammalian cells and has proven to be a useful tool for gene expression studies and gene therapy [124]. Epstein–Barr virus (EBV) is a herpes virus that replicates its genome episomally in host cells upon latent infection [125] by expressing Epstein–Barr nuclear antigen 1 (EBNA-1), which in turn recognizes the *oriP* site and initiates replication [125]. This system was exploited by encoding *oriP* and EBNA-1 onto a DNA vector for the long-term replication of plasmids in mesenchymal stem cells [126] and human fibroblasts [127]. Other viruses, such as SV40 and papillomaviruses, have similar systems with *trans*-acting elements that allow them to replicate in mammalian cells [128]. For a more detailed overview of the application of these viral sequences in episomal expression vectors, we refer the reader to articles by Van Craenenbroeck et al. [124] and Jackson et al. [128]. The generation of viral hybrid vector systems was an attempt to circumvent the safety concerns of viral vectors. Although there is potential for minimized vector systems to encode these viral sequences, thus turning them into minimized episomes, inclusion of these sequences would, again, result in increased vector length.

## 5. Therapeutic Needs Best Addressed by Minimized Vectors

### 5.1. DNA Vaccines

For DNA vaccines, antigens of interest are encoded on DNA vectors in an expression cassette. These vectors allow the expression of foreign proteins and cause subsequent formation of antibodies in the vaccinated host. In spite of the potential advantages afforded by plasmids for this endeavor, their development as DNA vaccine vectors was quickly hindered by their failure to elicit desired levels of immunogenic responses. Their failure was a consequence of poor transfection, transient antigen expression, and transgene silencing [129]. One of the first incentives to create minimal DNA vectors was for use as improved DNA vaccines. Because the presence of bacterial segments of 1,000 bp or more mediates transgene silencing in some tissues (reviewed by Williams [130]), more prolonged and sustained antigen expression can be achieved with minimized vectors than with plasmid vectors [70,130].

Linear contaminants or any closed circular molecules that break upon vaccine delivery increase the probability that DNA fragments may insert into chromosomes disadvantageously. Minimized DNA vectors, which are resistant to shear forces and thus not likely to break, represent the natural next step in vaccine development. In addition, because there are many more molecules of a smaller vector per mass than a larger vector, this means there are a higher number of expression cassettes per volume. Thus, an effective vaccine dose could be achieved in a smaller volume [27].

Three clinical trials have been performed to treat chronic hepatitis B virus (HBV) using a plasmid DNA vaccine expressing the S envelope protein of HBV. Two of these trials resulted in seroconversion in 50% of patients, and the third trial showed that combination therapy of the DNA vaccine with an antiviral drug was less efficacious in people of Asian descent than it was in people of Caucasian descent (reviewed in [131]). This result demonstrated proof-of-principle that plasmids could be used as DNA vaccines, but also revealed plenty of room for vector improvement. Despite numerous pre-clinical studies, only one plasmid vector vaccine has been approved for use in humans at this time [132]. Minimized vectors might ultimately have better utility. For example, Dietz et al. found that minicircles elicit a stronger antigen-specific T-cell response and gave greater protective immunity than plasmids did in a mouse model of listeriosis [97].

DNA vaccines are currently being pursued as a replacement for live attenuated virus vaccines for the seasonal influenza virus. DNA vectors can be administered intradermally, without a needle. One such polyvalent DNA influenza vaccine was recently successfully tested in rabbits and pigs, as is standard progression for influenza vaccine testing [133,134]. In these animal trials, the minimized antibiotic-free plasmid constructs (1,700 bp plus either a 1,778 bp hemagglutinin gene or a 1,413 bp neuraminidase gene [135]) were more efficacious than the traditional plasmid constructs (3,665 bp plus either the hemagglutinin or the neuraminidase gene) [134]. These DNA vaccines could be made even smaller with the minivector system (106 bp plus the antigenic sequences), which should further improve antigen gene delivery and expression.

### 5.2. Cancer and Immunotherapy

Plasmid vectors and, in at least one case, minimized DNA vectors, have been used successfully to deliver gene therapies against cancer (Table 1) [47,136,137,138]. In pre-clinical work to induce antitumor activity, Wu et al. used minicircles to induce the expression of interferon-γ in nasopharyngeal carcinoma cells. Expression of interferon-γ had a profound anti-proliferative effect in vitro and a survival-mediating antitumor effect in xenografted mice [139]. Minimal-size (MIDGE) vector systems have been used successfully both pre-clinically and clinically as DNA vaccines against advanced stage cancers in conjunction with double stem loop immunomodulator molecules (dSLIM) that serve as effective immune adjuvants [76,140,141]. MIDGE has also been tested for the sensitization of melanoma cells to chemotherapy [142]. Furthermore, in 2011, Zhao et al. showed the potential of minivectors encoding short hairpin RNA (shRNA) against anaplastic lymphoma kinase (ALK) [72]. They demonstrated increased transfection efficiency and gene silencing capability of minivectors compared to plasmid, and equivalence of gene silencing compared to siRNA. They found that minivectors transfect Jurkat cells, which are normally refractory to transfection, and were able to slow the growth of anaplastic large cell lymphoma cells in vitro using knockdown of ALK [72]. Minicircles were also recently used to engineer T-cells with *Sleeping Beauty* transposon [143] and were further used to deliver bi-specific antibodies, allowing T-cells to kill B-cell lymphomas [144]. Minicircles have also been incorporated into cancer detection systems, where they are used as tumor activators to facilitate detection of endogenous blood biomarkers [145]. Theoretically, minimized DNA vectors also could be used to restore expression of tumor suppression genes, silence transcripts of oncogenic proteins, or sensitize cells of the immune system against malignant cells.

### 5.3. Stem Cell Reprogramming

Minimized DNA vectors can be used to reprogram somatic cells for generation of induced pluripotent stem cells (iPSC). In 2010, Jia et al. created a 2A-linked polycistronic minicircle containing the genes for the reprogramming factors Lin28, Oct4, Sox2, and Nanog, with GFP as a reporter [146]. This group was able to induce pluripotency in human adipose stem cells. Although the rate of reprogramming was low compared to that of viruses, iPSC reprogrammed with minicircle DNA formed embryoid bodies in culture and teratomas in immune-deficient mice. They found minicircle efficiency was higher than that of plasmid DNA [146].

More recently, Daneshvar et al. created iPSCs from umbilical cord mesenchymal stem cells using minicircles with the same four reprogramming factors, but without the need for a layer of feeder cells supplying additional nutrients and reprogramming factors [147]. Fernandes and Chari engineered neural stem cells to secrete brain-derived neurotrophic factor (BDNF) using magnetic nanoparticles in conjunction with minicircle or plasmid DNA [148]. The minicircle had over five-fold higher transfection efficiency than the plasmid, and minicircle-transfected cells had a ten-fold increase in BDNF secretion over plasmid-transfected cells [148]. These pre-clinical tests demonstrate that stem cell reprogramming is feasible with minimized DNA vectors.

### 5.4. Therapy for the Lungs

Lungs are easily accessible via aerosolization [149,150]. Thus far, however, gene therapy approaches for pulmonary diseases, including cystic fibrosis, cancer, and asthma, have not been fully realized. Impenetrability of the mucosal layer in the lung to gene therapy vectors (viral or non-viral), and only limited and transient gene expression explain the difficulties encountered in prior attempts [151,152,153]. Minimized vector systems have the potential to deliver to lungs because naked DNA minivectors smaller than 1,200 bp can be aerosolized [71].

Minicircles have been effectively used both in vitro and in vivo to target lung epithelial cells with enhanced GFP (eGFP), firefly luciferase (Luc), or DNAH5, which encodes an outer dynein arm protein involved in primary ciliary dyskinesia [150]. Minicircles carrying these genes displayed higher levels of gene expression compared to plasmids [150]. Higher and more prolonged gene expression afforded by minicircles or minivectors reduces the number of therapeutic treatments needed. Fewer vector administrations reduces the potential for adaptive immune responses, such as are seen with the use of viral vectors or plasmids (reviewed by [154]). In this regard, reduction of CpGs in plasmid vectors or the use of CpG-free plasmids permits repeated delivery to lungs without causing toxicity or immunogenicity [155,156]. Minimized vectors such as minivectors do not contain CpG motifs (unless they are specifically added as part of the design) [73]. Furthermore, the nanosize (~5 nm × 5 nm × 45 nm) of supercoiled minivectors may prevent them from getting stuck in the mucosal layer, allowing them to penetrate cells and improve lung therapy outcomes. Together, these findings not only mean that minivectors could be used to deliver genes to address lung-specific diseases, but may also enable the lung to be used as a route into the body for the systemic delivery of therapeutic molecules into the bloodstream.

### 5.5. Cardiovascular Uses

One of the most significant health issues facing the world today is cardiovascular disease. The enormous potential as well as the particular challenges of using gene therapy for cardiovascular diseases were recently reviewed [157]. The pitfall of reduced gene expression of naked plasmid injections (a standard delivery technique for gene therapy of the heart) was addressed by comparing delivery of minicircle and plasmid encoding the same target [158]. Minicircle expressed similarly to plasmids [158], thereby constituting a safer alternative to plasmid vectors for treatment of heart diseases. Minicircles may be the better alternative to viral vectors because of viral-associated immunogenicity issues, as reported in a study aimed to treat familial hypercholesterolemia [159]. Finally, minicircles encoding either shRNA or miRNA also renew hope of combating heart disease with gene therapy [160,161], particularly because of their size advantage compared to other vectors, and especially when combined with short therapeutic sequences.

### 5.6. Dermal Uses

The skin is a readily accessible target for non-viral gene therapy. One application for gene therapy of the skin is wound healing in diabetics, specifically by enhancing expression of vascular endothelial growth factor (VEGF). VEGF triggers the growth of new blood vessels, bringing more healing factors to a wound. Yoon et al. enhanced wound healing in diabetic mice by subcutaneously injecting vectors expressing VEGF in a microbubble solution, then forcing transfection by popping the bubbles with ultrasound [162]. They found that a minicircle construct expressed VEGF more effectively than the plasmid constructs they tested [162].

DNA vector treatments can be applied for psoriasis, an autoimmune skin condition caused by chronic inflammation and dysregulated angiogenesis [163]. Expressing a gene that inhibits transforming growth factor-β prevents aberrant angiogenesis that can treat not only psoriasis [163] but also improve wound healing in the cornea [164]. The most logical method for delivering gene therapy vectors to the skin is topical application of a vector, which is theoretically possible. Subcutaneous injections would improve access of the vector to dermal immune cells. Using sonoporation to precisely release DNA vectors from microbubbles only at the wound or plaque psoriasis site prevents distant cells from being exposed to unwanted immune-stimulating or immune-suppressing genes.

## 6. Concluding Remarks: Moving Minimized Non-Viral DNA Vectors into the Clinic

Worldwide, the total number of reported active gene therapy clinical trials is 2,400 [165]. Non-viral DNA vectors—plasmids, oligonucleotides, and the pCOR, pORT and MIDGE systems—have been thus far used less frequently than viral vectors (21% vs 79%). Other non-viral vector systems described in this review, such as pFAR and minicircles, have been tested only pre-clinically. Some of these pre-clinical studies have been described in the reviews of Vandermeulen et al. [45], Gaspar et al. [9], and Wong et al. [115]. In this review, we addressed some of the specific minicircle studies that occurred since those reviewed by Gaspar et al. in 2015.

Table 1 provides the status of non-viral DNA vectors in clinical trials. So far, only pCOR, pORT, and MIDGE are listed in this table, but recent advancements made in minimized DNA vectors should increase these numbers. Numerous successful pre-clinical studies will lead to increased numbers of minimized DNA vectors in clinical trials for some of the most sought-after targets in gene therapy, like cancer and HIV. Efforts continue to make production of minimized DNA vectors more cost effective and to improve their purity [92,93,94,95].

Ideal for delivering short hairpin RNA [72,166], miRNA [160], cytokines [139], and other biologics, such as synthetic protein drugs [167,168], minimized DNA vectors allow for multiple important uses. Minicircles have even been co-delivered with drugs [169,170,171]. Additional future applications of minimized DNA vectors include engineering them to recognize specific cells or organs, construction of genetic logic gates to control gene expression [172,173], and the potential to control the shape of specific vectors [174]. To maintain the advantages of small vector size, delivery of long genes could be achieved by breaking such genes into multiple fragments and encoding each fragment on multiple vectors [172,175]. Gene fragmentation and protein reconstitution [176] are tools that can be easily incorporated into minimized DNA vectors. Furthermore, minimized DNA vectors are potentially better suited for the treatment of polygenic diseases because multiple vectors can be delivered simultaneously and administered repeatedly as needed.

In this review, we have highlighted numerous ways researchers have endeavored to realize the potential of gene therapy using non-viral DNA vectors. It is critically important that more work be done to understand the advantages and disadvantages of each tool in the gene therapy toolbox. Most of the advances made to non-viral DNA vector engineering have involved reducing length and removing problematic DNA sequences. Each of these advances, however, brought new problems to solve—problems of purity and scale. These new issues are only now beginning to be addressed; nevertheless, the steady progress and successes are encouraging. The improvements afforded by decreased cellular toxicity, increased transfection efficiency, increased number (moles) of payload delivered per mass, enhanced purity and yield, and decreased expense all combine to make non-viral DNA vectors a favorable gene therapy tool.

## Figures and Tables

**Table 1:** Non-replicating non-viral DNA vectors in gene therapy trials.

Non-Viral DNA Vector		Disease^1^ (*n*)		Phase
Plasmid (190 studies)	Monogenic	Cystic fibrosis (1)		1/2
	Polygenic	Cancer	T-cell immunotherapy (32)	1,2
			Therapeutic DNA vaccines (31)	1,2
			Other cancer treatments (32)	1,2
		Diabetes (1)		1,2
		Heart (13)		1,2
		Other^2^ (10)		1,2
	Infectious	Hepatitis B (5)		1,2
		Hepatitis C (3)		1,2
		HIV (52)		1,2
		HPV (2)		1,2
		Influenza (14)		1
		Malaria (2)		1
		Other^3^ (22)		1,2
Naked DNA (9 studies)	Monogenic	Von Willebrand disease (1)		-
	Polygenic	Cancer (2)		1/2
		Cancer vaccine (2)		1
	Infectious	Hepatitis B (2)		1,2
		HIV (3)		1
Oligonucleotide (141 studies)	Monogenic	Cystic fibrosis (2)		1,2
		Muscular dystrophy (4)		1,2
		Von Willebrand disease (2)		2
	Polygenic	Asthma (6)		1,2
		Cancer (79)		1–4
		Diabetes (3)		1,2
		Heart (5)		1,2
		Other^4^ (26)		1-3
	Infectious	Hepatitis B (3)		1,2
		Hepatitis C (1)		2
		HIV (3)		1,2
		Other^5^ (7)		1-3
pCOR^6^ (1 study)	Polygenic	Heart (1)		2
pORT^7^ (1 study)	Infectious	HIV (1)		1
MIDGE^8^ (1 study)	Polygenic	Cancer		1/2

Data were extracted from ClinicalTrials.gov on January 16, 2017 [23]. ^1^Examples of diseases with relevant advances in gene therapy [24](GeneTherapyNet.com); ^2^allergic rhinitis (*n* = 3), food allergy (*n* = 1), osteogenesis (*n* = 1), secondary Reynaud’s phenomenon (*n* = 1), arteriosclerosis (*n* = 3), bone tissue repair (*n* = 1); ^3^cytomegalovirus (*n* = 4), Ebola (*n* = 3), severe acute respiratory syndrome (SARS) (*n* = 1), West Nile fever (*n* = 1), Zika (*n* = 2), Dengue (*n* = 1), bacterial-related (*Escherichia coli*, *Klebsiella*, Enterobacteriacae) (*n* = 8), Middle East respiratory syndrome coronavirus (MERS CoV) (*n* = 1), genital herpes (*n* = 1); ^4^glaucoma (*n* = 2), allergic rhinitis (*n* = 2), rheumatoid arthritis (*n* = 1), shoulder stiffness (*n* = 1), ulcerative colitis (*n* = 2), triglycerides (*n* = 1), congenital malformation (*n* = 1), Crohn’s disease (*n* = 2), plaque psoriasis (*n* = 1), atherosclerosis (*n* = 1), mitochondrial disease (*n* = 1), schizophrenia (*n* = 1), obesity (*n* = 3), inflammatory diseases (*n* = 1), macular degeneration (*n* = 1), anemia (*n* = 2), eczema (*n* = 1), food allergies (*n* = 1); ^5^dermatophytes (*n* = 1), bacterial-related (gram negative bacteria) (*n* = 3), tuberculosis (*n* = 2), hookworm disease (*n* = 1); ^6^plasmids with conditional origin of replication; ^7^operator repressor titration plasmids; ^8^minimalistic immunologically defined gene expression. pCOR: plasmid with conditional origin of replication; pORT: operator repressor titration plasmid; MIDGE: minimalistic immunologically defined gene expression; HIV: human immunodeficiency virus; HPV: human papilloma virus.

**Table 2:** Vector production and gene therapy advantages and disadvantages of non-viral DNA vectors, with special emphasis on minimized vector systems.

Type of DNA vector	*ori*	AR	Advantages	Disadvantages	Reference
**Plasmid**	Yes	Yes	Safer than viruses and can be delivered more than once ^1^ Low risk of integration^1^ Can accommodate a long genetic payload Cheap, and easy to construct, produce, and store	DNA carrier/vehicle introduces additional complexity ^1^ Poor transfection efficiency naked CpG motifs can lead to inflammation and/or gene silencing Difficulty surrounding residual antibiotic and/or endotoxin	[47]
**AR-free miniplasmids**	Yes	No	Shorter than plasmids Lower metabolic burden on host strain	Still contains bacterial sequence which can lead to immune response and transgene silencing	[18,45,46]
pORT			Sequence addition to miniplasmid is short and selection does not involve gene expression Used in clinical trials as a DNA vaccine Easy to generate and produce	Mutant host strain required for propagation	[34,37,38,39,40,41,42,48,49,50]
pCOR			Narrow host range/low risk of spread No requirement for complex growth medium Used in clinical trials	Mutant host strain required for propagation tRNA expression required for selection	[35,44,51,52,53,54]
pFAR			Vector is only 1.1 kb The amber mutation used is very efficiently suppressed Growth medium does not contain animal-derived components Production process yields mostly monomeric closed circular DNA Both the plasmid and host strain are well-defined and described	Mutant host strain required for propagation tRNA expression required for selection	[36]
Post-segregational killing (PSK) systems			Sequence addition to miniplasmid is short Toxin is highly efficient for selection	PSK genes can cause mild toxicity	[55,56,57]
RNA IN/RNA OUT			RNA sequence is only transcribed in prokaryotes	Mutant host strain required for propagation	[58,59]
RNA I/RNA II			No sequence addition to plasmid necessary Can be produced in gram quantities more easily than standard plasmids	Mutant host strain required for propagation	[60,61]
Overexpression systems			No mutant host strain needed	Not much shorter than plasmids Potential metabolic burden on host strain Possible antibiotic contamination of final product	[62,63]
**Circular Covalently Closed Vectors**	No	No	Enhanced transfection and persistence in vitro and in vivo Little to no bacterial sequence	High production costs relative to plasmids Potential issues with scaling for clinical use	[9,18,32,64]
Minicircle			Vectors have been designed that are appropriate for mammalian mitochondrial gene therapy	Some plasmid and other DNA contaminants can remain in the final product	[27,65,66,67,68,69,70]
Minivector			Smallest circular DNA vector Most supercoiled DNA vector Greatest purity Naked minivector <1200 bp resists nebulization shear forces	Not well-known in the field	[33,71,72,73]
Miniknot			Potentially superior compactness Potentially resistant to nicking Potentially valuable for forceful administration procedures (e.g., aerosolization, jet-injection, etc.)	In hypothesis stage	[74]
**Linear Covalently Closed Vectors (“dumbbell-shaped”)**	No	No	Decreased risk of negative genome insertion events and nuclease attack because of protected ends Some systems are just the expression cassette alone	Vectors do not exhibit normal supercoiling, possessing only the qualities of linear DNA	
MIDGE			Smallest expression vectors reported Used in phase 1−2 clinical trials as a DNA vaccine Can be chemically modified to allow targeting	Potential rapid clearance Costly and time-consuming production	[75,76,77,78,79,80,81,82,83,84]
MiLV			Production process avoids bacterial contaminants such as endotoxin	PCR amplification of product is potentially error prone	[85]
Ministring			Chromosomal integration causes apoptotic cell death One-step in vivo production system This system can also create ccc vector topology	Uses ampicillin resistance as a selection marker Residual ampicillin or endotoxin possible in final product	[86,87,88,89]
**Mini-intronic plasmid**	Yes^2^	No	Production process and yield is the same as standard plasmids, but subsequent splicing removes problematic sequences Inclusion of introns is reported to enhance transgene expression Uses RNA IN/RNA OUT as a selectable marker system Reported to have higher transgene expression levels than plasmids or minicircles	Vector length is not as reduced as some minimal systems, requiring the use of more transfection reagent Small chance of unspliced or mis-spliced mRNA Possible immune recognition of *ori* or other bacterial sequences before splicing occurs	[90]

*ori* : origin of replication; AR: antibiotic resistance; CpG: cytosine-phosphate-guanine dinucleotide; pORT: operator repressor titration plasmids; pCOR: plasmids with conditional origin of replication; pFAR: plasmids free of antibiotic resistance; tRNA: transfer RNA; PSK: post-segregational killing; MIDGE: minimalistic immunologically defined gene expression; MiLV: micro-linear vector; PCR: polymerase chain reaction; ^1^True of most if not all non-viral DNA vectors; ^2^Mini-intronic plasmids are produced as standard plasmids and initially contain the same elements when transfection takes place. Only after splicing has occurred inside the target cell are undesirable elements such as *ori* finally removed.

## References

[1] Rao R.C., Zacks D.N. (2014). Cell and gene therapy. Dev. Ophthalmol..

[2] Cooney A.L., Abou Alaiwa M.H., Shah V.S., Bouzek D.C., Stroik M.R., Powers L.S., Gansemer N.D., Meyerholz D.K., Welsh M.J., Stoltz D.A. (2016). Lentiviral-mediated phenotypic correction of cystic fibrosis pigs. JCI Insight.

[3] Benjamin R., Berges B.K., Solis-Leal A., Igbinedion O., Strong C.L., Schiller M.R. (2016). TALEN gene editing takes aim on HIV. Hum. Genet..

[4] Samson S.L., Gonzalez E.V., Yechoor V., Bajaj M., Oka K., Chan L. (2008). Gene therapy for diabetes: metabolic effects of helper-dependent adenoviral exendin 4 expression in a diet-induced obesity mouse model. Mol. Ther. J. Am. Soc. Gene Ther..

[5] Bicho D., Queiroz J.A., Tomaz C.T. (2015). Influenza plasmid DNA vaccines: Progress and prospects. Curr. Gene Ther..

[6] Al-Dosari M.S., Gao X. (2009). Nonviral gene delivery: Principle, limitations, and recent progress. AAPS J..

[7] Jones C.H., Hill A., Chen M., Pfeifer B.A. (2015). Contemporary approaches for nonviral gene therapy. Discov. Med..

[8] Keles E., Song Y., Du D., Dong W.-J., Lin Y. (2016). Recent progress in nanomaterials for gene delivery applications. Biomater. Sci..

[9] Gaspar V., de Melo-Diogo D., Costa E., Moreira A., Queiroz J., Pichon C., Correia I., Sousa F. (2015). Minicircle DNA vectors for gene therapy: Advances and applications. Expert Opin. Biol. Ther..

[10] Hacein-Bey-Abina S., de Saint Basile G., Cavazzana-Calvo M. (2003). Gene therapy of X-linked severe combined immunodeficiency. Methods Mol. Biol. Clifton NJ.

[11] Hacein-Bey-Abina S., von Kalle C., Schmidt M., Le Deist F., Wulffraat N., McIntyre E., Radford I., Villeval J.-L., Fraser C.C., Cavazzana-Calvo M., Fischer A. (2003). A serious adverse event after successful gene therapy for X-linked severe combined immunodeficiency. N. Engl. J. Med..

[12] Loring H.S., ElMallah M.K., Flotte T.R. (2016). Development of rAAV2-CFTR: History of the First rAAV Vector Product to be Used in Humans. Hum. Gene Ther. Methods.

[13] Wold W.S.M., Toth K. (2013). Adenovirus vectors for gene therapy, vaccination and cancer gene therapy. Curr. Gene Ther..

[14] Thomas C.E., Ehrhardt A., Kay M. (2003). A Progress and problems with the use of viral vectors for gene therapy. Nat. Rev. Genet..

[15] Kreppel F., Kochanek S. (2008). Modification of adenovirus gene transfer vectors with synthetic polymers: A scientific review and technical guide. Mol. Ther. J. Am. Soc. Gene Ther..

[16] Sinn P.L., Burnight E.R., McCray P.B. (2009). Progress and Prospects: Prospects of repeated pulmonary administration of viral vectors. Gene Ther..

[17] Lesch H.P., Pikkarainen J.T., Kaikkonen M.U., Taavitsainen M., Samaranayake H., Lehtolainen-Dalkilic P., Vuorio T., Määttä A.-M., Wirth T., Airenne K.J. (2009). Avidin fusion protein-expressing lentiviral vector for targeted drug delivery. Hum. Gene Ther..

[18] Schleef M. (2013). Minicircle and Miniplasmid DNA Vectors: The Future of Non-Viral and Viral Gene Transfer.

[19] Kormann M.S.D., Hasenpusch G., Aneja M.K., Nica G., Flemmer A.W., Herber-Jonat S., Huppmann M., Mays L.E., Illenyi M., Schams A. (2011). Expression of therapeutic proteins after delivery of chemically modified mRNA in mice. Nat. Biotechnol..

[20] Yin H., Kanasty R.L., Eltoukhy A.A., Vegas A.J., Dorkin J.R., Anderson D.G. (2014). Non-viral vectors for gene-based therapy. Nat. Rev. Genet..

[21] Wolff J.A., Malone R.W., Williams P., Chong W., Acsadi G., Jani A., Felgner P.L. (1990). Direct gene transfer into mouse muscle in vivo. Science.

[22] Izsvák Z., Chuah M.K.L., Vandendriessche T., Ivics Z. (2009). Efficient stable gene transfer into human cells by the Sleeping Beauty transposon vectors. Methods San Diego Calif..

[23] Home - ClinicalTrials.gov. https://clinicaltrials.gov/ct2/home.

[24] New and Updated Clinical Gene Therapy Trials. http://www.genetherapynet.com/clinicaltrialsgov.html.

[25] Rodriguez E. (2004). Nonviral DNA vectors for immunization and therapy: Design and methods for their obtention. J. Mol. Med..

[26] Mairhofer J., Grabherr R. (2008). Rational vector design for efficient non-viral gene delivery: Challenges facing the use of plasmid DNA. Mol. Biotechnol..

[27] Stenler S., Blomberg P., Smith C.I.E. (2014). Safety and efficacy of DNA vaccines: Plasmids vs. minicircles. Hum. Vaccines Immunother..

[28] Lu J., Zhang F., Xu S., Fire A.Z., Kay M.A. (2012). The extragenic spacer length between the 5’ and 3’ ends of the transgene expression cassette affects transgene silencing from plasmid-based vectors. Mol. Ther. J. Am. Soc. Gene Ther..

[29] Faurez F., Dory D., Le Moigne V., Gravier R., Jestin A. (2010). Biosafety of DNA vaccines: New generation of DNA vectors and current knowledge on the fate of plasmids after injection. Vaccine.

[30] Ahmad-Nejad P., Häcker H., Rutz M., Bauer S., Vabulas R.M., Wagner H. (2002). Bacterial CpG-DNA and lipopolysaccharides activate Toll-like receptors at distinct cellular compartments. Eur. J. Immunol..

[31] Kreiss P., Cameron B., Rangara R., Mailhe P., Aguerre-Charriol O., Airiau M., Scherman D., Crouzet J., Pitard B. (1999). Plasmid DNA size does not affect the physicochemical properties of lipoplexes but modulates gene transfer efficiency. Nucleic Acids Res..

[32] Stenler S., Wiklander O.P., Badal-Tejedor M., Turunen J., Nordin J.Z., Hallengärd D., Wahren B., El Andaloussi S., Rutland M.W., Smith C.E. (2014). Micro-minicircle gene therapy: Implications of size on fermentation, complexation, shearing resistance, and expression. Mol. Ther. Acids.

[33] Hornstein B.D., Roman D., Arévalo-Soliz L.M., Engevik M.A., Zechiedrich L. (2016). Effects of circular DNA length on transfection efficiency by electroporation into HeLa cells. PLoS ONE.

[34] Cranenburgh R.M., Hanak J.A., Williams S.G., Sherratt D.J. (2001). *Escherichia coli* strains that allow antibiotic-free plasmid selection and maintenance by repressor titration. Nucleic Acids Res..

[35] Soubrier F., Cameron B., Manse B., Somarriba S., Dubertret C., Jaslin G., Jung G., Caer C.L., Dang D., Mouvault J.M. (1999). pCOR: A new design of plasmid vectors for nonviral gene therapy. Gene Ther..

[36] Marie C., Vandermeulen G., Quiviger M., Richard M., Préat V., Scherman D. (2010). pFARs, plasmids free of antibiotic resistance markers, display high-level transgene expression in muscle, skin and tumour cells. J. Gene Med..

[37] Mwau M., Cebere I., Sutton J., Chikoti P., Winstone N., Wee E.G.-T., Beattie T., Chen Y.-H., Dorrell L., McShane H. (2004). A human immunodeficiency virus 1 (HIV-1) clade A vaccine in clinical trials: Stimulation of HIV-specific T-cell responses by DNA and recombinant modified vaccinia virus Ankara (MVA) vaccines in humans. J. Gen. Virol..

[38] Ramos I., Alonso A., Peris A., Marcen J.M., Abengozar M.A., Alcolea P.J., Castillo J.A., Larraga V. (2009). Antibiotic resistance free plasmid DNA expressing LACK protein leads towards a protective Th1 response against Leishmania infantum infection. Vaccine.

[39] Saubi N., Mbewe-Mvula A., Gea-Mallorqui E., Rosario M., Gatell J.M., Hanke T., Joseph J. (2012). Pre-clinical development of BCG.HIVA(CAT), an antibiotic-free selection strain, for HIV-TB pediatric vaccine vectored by lysine auxotroph of BCG. PLoS ONE.

[40] Garmory H.S., Leckenby M.W., Griffin K.F., Elvin S.J., Taylor R.R., Hartley M.G., Hanak J.A.J., Williamson E.D., Cranenburgh R.M. (2005). Antibiotic-free plasmid stabilization by operator-repressor titration for vaccine delivery by using live *Salmonella enterica* Serovar typhimurium. Infect. Immun..

[41] Leckenby M.W., Spear A.M., Neeson B.N., Williamson E.D., Cranenburgh R.M., Atkins H.S. (2009). Enhanced vaccine antigen delivery by *Salmonella* using antibiotic-free operator-repressor titration-based plasmid stabilisation compared to chromosomal integration. Microb. Pathog..

[42] Huang J.-M., Sali M., Leckenby M.W., Radford D.S., Huynh H.A., Delogu G., Cranenburgh R.M., Cutting S.M. (2010). Oral delivery of a DNA vaccine against tuberculosis using operator-repressor titration in a *Salmonella enterica* vector. Vaccine.

[43] Lilly J., Camps M. (2015). Mechanisms of theta plasmid replication. Microbiol. Spectr..

[44] Nikol S., Baumgartner I., Van Belle E., Diehm C., Visoná A., Capogrossi M.C., Ferreira-Maldent N., Gallino A., Wyatt M.G., Wijesinghe L.D. (2008). TALISMAN 201 investigators Therapeutic angiogenesis with intramuscular NV1FGF improves amputation-free survival in patients with critical limb ischemia. Mol. Ther. J. Am. Soc. Gene Ther..

[45] Vandermeulen G., Marie C., Scherman D., Préat V. (2011). New generation of plasmid backbones devoid of antibiotic resistance marker for gene therapy trials. Mol. Ther..

[46] Oliveira P.H., Mairhofer J. (2013). Marker-free plasmids for biotechnological applications—Implications and perspectives. Trends Biotechnol..

[47] Bathula S.R., Huang L., Abraham D.J. (2003). Gene Therapy with Plasmid DNA. Burger’s Medicinal Chemistry and Drug Discovery.

[48] Williams S.G., Cranenburgh R.M., Weiss A.M., Wrighton C.J., Sherratt D.J., Hanak J.A. (1998). Repressor titration: A novel system for selection and stable maintenance of recombinant plasmids. Nucleic Acids Res..

[49] Cranenburgh R.M., Lewis K.S., Hanak J.A.J. (2004). Effect of plasmid copy number and *lac* operator sequence on antibiotic-free plasmid selection by operator-repressor titration in *Escherichia coli*. J. Mol. Microbiol. Biotechnol..

[50] Durany O., Bassett P., Weiss A.M.E., Cranenburgh R.M., Ferrer P., López-Santín J., de Mas C., Hanak J.A.J. (2005). Production of fuculose-1-phosphate aldolase using operator-repressor titration for plasmid maintenance in high cell density *Escherichia coli* fermentations. Biotechnol. Bioeng..

[51] Kornowski R., Fuchs S., Epstein S.E., Branellec D., Schwartz B. (2000). Catheter-based plasmid-mediated transfer of genes into ischemic myocardium using the pCOR plasmid. Coron. Artery Dis..

[52] Soubrier F., Laborderie B., Cameron B. (2005). Improvement of pCOR plasmid copy number for pharmaceutical applications. Appl. Microbiol. Biotechnol..

[53] Witzenbichler B., Mahfoudi A., Soubrier F., Le Roux A., Branellec D., Schultheiss H.-P., Isner J.M. (2006). Intramuscular gene transfer of fibroblast growth factor-1 using improved pCOR plasmid design stimulates collateral formation in a rabbit ischemic hindlimb model. J. Mol. Med. Berl. Ger..

[54] Maulik N. (2009). NV1FGF, a pCOR plasmid-based angiogenic gene therapy for the treatment of intermittent claudication and critical limb ischemia. Curr. Opin. Investig. Drugs Lond. Engl. 2000.

[55] Van Melderen L. (2002). Molecular interactions of the CcdB poison with its bacterial target, the DNA gyrase. Int. J. Med. Microbiol. IJMM.

[56] Szpirer C.Y., Milinkovitch M.C. (2005). Separate-component-stabilization system for protein and DNA production without the use of antibiotics. BioTechniques.

[57] Peubez I., Chaudet N., Mignon C., Hild G., Husson S., Courtois V., De Luca K., Speck D., Sodoyer R. (2010). Antibiotic-free selection in *E. coli*: New considerations for optimal design and improved production. Microb. Cell Factories.

[58] Luke J., Carnes A.E., Hodgson C.P., Williams J.A. (2009). Improved antibiotic-free DNA vaccine vectors utilizing a novel RNA based plasmid selection system. Vaccine.

[59] Luke J.M., Carnes A.E., Williams J.A. (2014). Development of antibiotic-free selection system for safer DNA vaccination. Methods Mol. Biol. Clifton NJ.

[60] Pfaffenzeller I., Mairhofer J., Striedner G., Bayer K., Grabherr R. (2006). Using ColE1-derived RNA I for suppression of a bacterially encoded gene: Implication for a novel plasmid addiction system. Biotechnol. J..

[61] Mairhofer J., Cserjan-Puschmann M., Striedner G., Nöbauer K., Razzazi-Fazeli E., Grabherr R. (2010). Marker-free plasmids for gene therapeutic applications—Lack of antibiotic resistance gene substantially improves the manufacturing process. J. Biotechnol..

[62] Xu H.H., Real L., Bailey M.W. (2006). An Array of *Escherichia coli* clones over-expressing essential proteins: A new strategy of identifying cellular targets of potent antibacterial compounds. Biochem. Biophys. Res. Commun..

[63] Goh S., Good L. (2008). Plasmid selection in *Escherichia coli* using an endogenous essential gene marker. BMC Biotechnol..

[64] Šimčíková M., Prather K.L.J., Prazeres D.M.F., Monteiro G.A. (2015). Towards effective non-viral gene delivery vector. Biotechnol. Genet. Eng. Rev..

[65] Darquet A.M., Cameron B., Wils P., Scherman D., Crouzet J. (1997). A new DNA vehicle for nonviral gene delivery: Supercoiled minicircle. Gene Ther..

[66] Kreiss P., Cameron B., Darquet A.M., Scherman D., Crouzet J. (1998). Production of a new DNA vehicle for gene transfer using site-specific recombination. Appl. Microbiol. Biotechnol..

[67] Darquet A.M., Rangara R., Kreiss P., Schwartz B., Naimi S., Delaere P., Crouzet J., Scherman D. (1999). Minicircle: An improved DNA molecule for in vitro and in vivo gene transfer. Gene Ther..

[68] Bigger B.W., Tolmachov O., Collombet J.M., Fragkos M., Palaszewski I., Coutelle C. (2001). An *araC*-controlled bacterial cre expression system to produce DNA minicircle vectors for nuclear and mitochondrial gene therapy. J. Biol. Chem..

[69] Gaspar V.M., Maia C.J., Queiroz J.A., Pichon C., Correia I.J., Sousa F. (2014). Improved minicircle DNA biosynthesis for gene therapy applications. Hum. Gene Ther. Methods.

[70] Schleef M., Schirmbeck R., Reiser M., Michel M.-L., Schmeer M. (2015). Minicircle: Next generation DNA vectors for vaccination. Methods Mol. Biol. Clifton NJ.

[71] Catanese D.J., Fogg J.M., Schrock D.E., Gilbert B.E., Zechiedrich L. (2012). Supercoiled minivector DNA resists shear forces associated with gene therapy delivery. Gene Ther..

[72] Zhao N., Fogg J.M., Zechiedrich L., Zu Y. (2011). Transfection of shRNA-encoding Minivector DNA of a few hundred base pairs to regulate gene expression in lymphoma cells. Gene Ther..

[73] Fogg J.M., Kolmakova N., Rees I., Magonov S., Hansma H., Perona J.J., Zechiedrich E.L. (2006). Exploring writhe in supercoiled minicircle DNA. J. Phys. Condens. Matter Inst. Phys. J..

[74] Tolmachov O.E. (2010). Tightly-wound miniknot vectors for gene therapy: A potential improvement over supercoiled minicircle DNA. Med. Hypotheses.

[75] Schirmbeck R., König-Merediz S.A., Riedl P., Kwissa M., Sack F., Schroff M., Junghans C., Reimann J., Wittig B. (2001). Priming of immune responses to hepatitis B surface antigen with minimal DNA expression constructs modified with a nuclear localization signal peptide. J. Mol. Med. Berl. Ger..

[76] Wittig B., Märten A., Dorbic T., Weineck S., Min H., Niemitz S., Trojaneck B., Flieger D., Kruopis S., Albers A. (2001). Therapeutic vaccination against metastatic carcinoma by expression-modulated and immunomodified autologous tumor cells: A first clinical phase I/II trial. Hum. Gene Ther..

[77] Schakowski F., Gorschlüter M., Junghans C., Schroff M., Buttgereit P., Ziske C., Schöttker B., König-Merediz S.A., Sauerbruch T., Wittig B. (2001). A novel minimal-size vector (MIDGE) improves transgene expression in colon carcinoma cells and avoids transfection of undesired DNA. Mol. Ther. J. Am. Soc. Gene Ther..

[78] López-Fuertes L., Pérez-Jiménez E., Vila-Coro A.J., Sack F., Moreno S., Konig S.A., Junghans C., Wittig B., Timón M., Esteban M. (2002). DNA vaccination with linear minimalistic (MIDGE) vectors confers protection against *Leishmania* major infection in mice. Vaccine.

[79] Moreno S., López-Fuertes L., Vila-Coro A.J., Sack F., Smith C.A., Konig S.A., Wittig B., Schroff M., Juhls C., Junghans C., Timón M. (2004). DNA immunisation with minimalistic expression constructs. Vaccine.

[80] Schakowski F., Gorschlüter M., Buttgereit P., Märten A., Lilienfeld-Toal M.V., Junghans C., Schroff M., König-Merediz S.A., Ziske C., Strehl J. (2007). Minimal size MIDGE vectors improve transgene expression in vivo. Vivo Athens Greece.

[81] Endmann A., Baden M., Weisermann E., Kapp K., Schroff M., Kleuss C., Wittig B., Juhls C. (2010). Immune response induced by a linear DNA vector: Influence of dose, formulation and route of injection. Vaccine.

[82] Galling N., Kobelt D., Aumann J., Schmidt M., Wittig B., Schlag P.M., Walther W. (2012). Intratumoral dispersion, retention, systemic biodistribution, and clearance of a small-size tumor necrosis factor-α-expressing MIDGE vector after nonviral in vivo jet-injection gene transfer. Hum. Gene Ther. Methods.

[83] Chen X., Xu X., Peng X., Jiang W., Yao L. (2015). Construction of PPENK-MIDGE-NLS gene vector and the expression in rat. Sheng Wu Gong Cheng Xue Bao Chin. J. Biotechnol..

[84] Jiang X., Yu H., Teo C.R., Tan G.S.X., Goh S.C., Patel P., Chua Y.K., Hameed N.B.S., Bertoletti A., Patzel V. (2016). Advanced design of dumbbell-shaped genetic minimal vectors improves non-coding and coding RNA expression. Mol. Ther..

[85] Wang H.-S., Chen Z.-J., Zhang G., Ou X.-L., Yang X.-L., Wong C.K.C., Giesy J.P., Du J., Chen S.-Y. (2012). A novel micro-linear vector for in vitro and in vivo gene delivery and its application for EBV positive tumors. PLoS ONE.

[86] Nafissi N., Slavcev R. (2012). Construction and characterization of an in vivo linear covalently closed DNA vector production system. Microb. Cell Factories.

[87] Nafissi N., Alqawlaq S., Lee E.A., Foldvari M., Spagnuolo P.A., Slavcev R.A. (2014). DNA ministrings: Highly safe and effective gene delivery vectors. Mol. Ther. Nucleic Acids.

[88] Sum C.H., Nafissi N., Slavcev R.A., Wettig S. (2015). Physical characterization of gemini surfactant-based synthetic vectors for the delivery of linear covalently closed (LCC) DNA ministrings. PLoS ONE.

[89] Wong S., Lam P., Nafissi N., Denniss S., Slavcev R. (2016). Production of double-stranded DNA ministrings. J. Vis. Exp. JoVE.

[90] Lu J., Zhang F., Kay M.A. (2013). A Mini-intronic Plasmid (MIP): A novel robust transgene expression vector in vivo and in vitro. Mol. Ther..

[91] Chen Z.-Y., Riu E., He C.-Y., Xu H., Kay M.A. (2008). Silencing of episomal transgene expression in liver by plasmid bacterial backbone DNA is independent of CpG methylation. Mol. Ther..

[92] Hou X.H., Guo X.Y., Chen Y., He C.-Y., Chen Z.-Y. (2015). Increasing the minicircle DNA purity using an enhanced triplex DNA technology to eliminate DNA contaminants. Mol. Ther. Methods Clin. Dev..

[93] Alves C.P.A., Šimčíková M., Brito L., Monteiro G.A., Prazeres D.M.F. (2016). Development of a nicking endonuclease-assisted method for the purification of minicircles. J. Chromatogr. A.

[94] Diamantino T., Pereira P., Queiroz J.A., Sousa Â., Sousa F. (2016). Minicircle DNA purification using a CIM^®^ DEAE-1 monolithic support: Liquid chromatography. J. Sep. Sci..

[95] Šimčíková M., Alves C.P.A., Brito L., Prather K.L.J., Prazeres D.M.F., Monteiro G.A. (2016). Improvement of DNA minicircle production by optimization of the secondary structure of the 5′-UTR of ParA resolvase. Appl. Microbiol. Biotechnol..

[96] Zhang C., Liu H., Gao S., Huang W., Wang Z. (2010). Polyethylenimine and minicircle DNA based gene transfer. Sheng Wu Gong Cheng Xue Bao Chin. J. Biotechnol..

[97] Dietz W.M., Skinner N.E.B., Hamilton S.E., Jund M.D., Heitfeld S.M., Litterman A.J., Hwu P., Chen Z.-Y., Salazar A.M., Ohlfest J.R. (2013). Minicircle DNA is superior to plasmid DNA in eliciting antigen-specific CD8+ T-cell responses. Mol. Ther..

[98] Remaut K., Sanders N.N., Fayazpour F., Demeester J., De Smedt S.C. (2006). Influence of plasmid DNA topology on the transfection properties of DOTAP/DOPE lipoplexes. J. Control. Release Off. J. Control. Release Soc..

[99] Bodine T., Catanese D.J. Jr., Arevalo-Soliz L.M., Fogg J.M., Zechiedrich L. Effect of size and topology on DNA vector survival in human serum.

[100] Munkonge F.M., Dean D.A., Hillery E., Griesenbach U., Alton E.W.F.W. (2003). Emerging significance of plasmid DNA nuclear import in gene therapy. Adv. Drug Deliv. Rev..

[101] Brandén L.J., Mohamed A.J., Smith C.I. (1999). A peptide nucleic acid-nuclear localization signal fusion that mediates nuclear transport of DNA. Nat. Biotechnol..

[102] Zanta M.A., Belguise-Valladier P., Behr J.P. (1999). Gene delivery: A single nuclear localization signal peptide is sufficient to carry DNA to the cell nucleus. Proc. Natl. Acad. Sci. USA.

[103] Vaysse L., Gregory L.G., Harbottle R.P., Perouzel E., Tolmachov O., Coutelle C. (2006). Nuclear-targeted minicircle to enhance gene transfer with non-viral vectors in vitro and in vivo. J. Gene Med..

[104] Song Y.K., Liu F., Liu D. (1998). Enhanced gene expression in mouse lung by prolonging the retention time of intravenously injected plasmid DNA. Gene Ther..

[105] Krieg A.M. (2002). Cpg motifs in bacterial DNA and their immune effects. Annu. Rev. Immunol..

[106] Vollmer J., Krieg A.M. (2009). Immunotherapeutic applications of CpG oligodeoxynucleotide TLR9 agonists. Adv. Drug Deliv. Rev..

[107] Krieg A.M., Yi A.K., Matson S., Waldschmidt T.J., Bishop G.A., Teasdale R., Koretzky G.A., Klinman D.M. (1995). CpG motifs in bacterial DNA trigger direct B-cell activation. Nature.

[108] Martinez-Alonso S., Martinez-Lopez A., Estepa A., Cuesta A., Tafalla C. (2011). The introduction of multi-copy CpG motifs into an antiviral DNA vaccine strongly up-regulates its immunogenicity in fish. Vaccine.

[109] Li J., Shi J.-L., Wu X.-Y., Fu F., Yu J., Yuan X.-Y., Peng Z., Cong X.-Y., Xu S.-J., Sun W.-B. (2015). Improvement of the immunogenicity of porcine circovirus type 2 DNA vaccine by recombinant ORF2 gene and CpG motifs. Viral Immunol..

[110] Guo X., Zhang Q., Hou S., Zhai G., Zhu H., Sánchez-Vizcaíno J.M. (2011). Plasmid containing CpG motifs enhances the efficacy of porcine reproductive and respiratory syndrome live attenuated vaccine. Vet. Immunol. Immunopathol..

[111] Bode C., Zhao G., Steinhagen F., Kinjo T., Klinman D.M. (2011). CpG DNA as a vaccine adjuvant. Expert Rev. Vaccines.

[112] Scheiermann J., Klinman D.M. (2014). Clinical evaluation of CpG oligonucleotides as adjuvants for vaccines targeting infectious diseases and cancer. Vaccine.

[113] Wang Q., Jiang W., Chen Y., Liu P., Sheng C., Chen S., Zhang H., Pan C., Gao S., Huang W. (2014). In vivo electroporation of minicircle DNA as a novel method of vaccine delivery to enhance HIV-1-specific immune responses. J. Virol..

[114] Mirkovitch J., Mirault M.-E., Laemmli U.K. (1984). Organization of the higher-order chromatin loop: Specific DNA attachment sites on nuclear scaffold. Cell.

[115] Wong S.P., Argyros O., Harbottle R.P. (2015). Sustained expression from DNA vectors. Adv. Genet..

[116] Patrushev L.I., Kovalenko T.F. (2014). Functions of noncoding sequences in mammalian genomes. Biochem. Biokhimiia.

[117] Argyros O., Wong S.P., Fedonidis C., Tolmachov O., Waddington S.N., Howe S.J., Niceta M., Coutelle C., Harbottle R.P. (2011). Development of S/MAR minicircles for enhanced and persistent transgene expression in the mouse liver. J. Mol. Med. Berl. Ger..

[118] Keaton M.A., Taylor C.M., Layer R.M., Dutta A. (2011). Nuclear scaffold attachment sites within ENCODE regions associate with actively transcribed genes. PLoS ONE.

[119] Verghese S.C., Goloviznina N.A., Skinner A.M., Lipps H.J., Kurre P. (2014). S/MAR sequence confers long-term mitotic stability on non-integrating lentiviral vector episomes without selection. Nucleic Acids Res..

[120] Xu Z., Chen F., Zhang L., Lu J., Xu P., Liu G., Xie X., Mu W., Wang Y., Liu D. (2016). Non-integrating lentiviral vectors based on the minimal S/MAR sequence retain transgene expression in dividing cells. Sci. China Life Sci..

[121] Jin C., Fotaki G., Ramachandran M., Nilsson B., Essand M., Yu D. (2016). Safe engineering of CAR T cells for adoptive cell therapy of cancer using long-term episomal gene transfer. EMBO Mol. Med..

[122] Koirala A., Conley S.M., Naash M.I. (2014). Episomal maintenance of S/MAR-containing non-viral vectors for RPE-based diseases. Adv. Exp. Med. Biol..

[123] Nehlsen K., Broll S., Bode J. (2006). Replicating minicircles: Generation of nonviral episomes for the efficient modification of dividing cells. Gene Ther Mol Biol.

[124] Van Craenenbroeck K., Vanhoenacker P., Haegeman G. (2000). Episomal vectors for gene expression in mammalian cells. Eur. J. Biochem..

[125] Leight E.R., Sugden B. (2000). EBNA-1: A protein pivotal to latent infection by Epstein-Barr virus. Rev. Med. Virol..

[126] Ali Hosseini Rad S.M., Bamdad T., Arefian E., Mossahebi-Mohammadi M., Sadeghizadeh M. (2015). An EBV-based plasmid can replicate and maintain in stem cells. Biotechnol. Prog..

[127] Drozd A.M., Walczak M.P., Piaskowski S., Stoczynska-Fidelus E., Rieske P., Grzela D.P. (2015). Generation of human iPSCs from cells of fibroblastic and epithelial origin by means of the *oriP*/EBNA-1 episomal reprogramming system. Stem Cell Res. Ther..

[128] Jackson D.A., Juranek S., Lipps H.J. (2006). Designing nonviral vectors for efficient gene transfer and long-term gene expression. Mol. Ther..

[129] Geiben-Lynn R., Greenland J.R., Frimpong-Boateng K., van Rooijen N., Hovav A.-H., Letvin N.L. (2008). CD4+ T lymphocytes mediate in vivo clearance of plasmid DNA vaccine antigen expression and potentiate CD8+ T-cell immune responses. Blood.

[130] Williams J.A. (2014). Improving DNA vaccine performance through vector design. Curr. Gene Ther..

[131] Ghasemi F., Rostami S., Ghayour-Mobarhan M., Meshkat Z. (2016). Current progress in the development of therapeutic vaccines for chronic hepatitis B virus infection. Iran. J. Basic Med. Sci..

[132] Halstead S.B., Thomas S.J. (2011). New Japanese encephalitis vaccines: Alternatives to production in mouse brain. Expert Rev. Vaccines.

[133] Borggren M., Nielsen J., Karlsson I., Dalgaard T.S., Trebbien R., Williams J.A., Fomsgaard A. (2016). A polyvalent influenza DNA vaccine applied by needle-free intradermal delivery induces cross-reactive humoral and cellular immune responses in pigs. Vaccine.

[134] Borggren M., Nielsen J., Bragstad K., Karlsson I., Krog J.S., Williams J.A., Fomsgaard A. (2015). Vector optimization and needle-free intradermal application of a broadly protective polyvalent influenza A DNA vaccine for pigs and humans. Hum. Vaccines Immunother..

[135] The Influenza Virus: Structure and Replication—Article in Motion. http://www.rapidreferenceinfluenza.com/chapter/B978-0-7234-3433-7.50009-8/aim/influenza-virus-structure.

[136] Sousa F., Passarinha L., Queiroz J.A. (2009). Biomedical application of plasmid DNA in gene therapy: A new challenge for chromatography. Biotechnol. Genet. Eng. Rev..

[137] Gorecki D.C. (2009). “Dressed-up” naked plasmids: Emerging vectors for non-viral gene therapy. Discov. Med..

[138] Williams P.D., Kingston P.A. (2011). Plasmid-mediated gene therapy for cardiovascular disease. Cardiovasc. Res..

[139] Wu J., Xiao X., Zhao P., Xue G., Zhu Y., Zhu X., Zheng L., Zeng Y., Huang W. (2006). Minicircle-IFNgamma induces antiproliferative and antitumoral effects in human nasopharyngeal carcinoma. Clin. Cancer Res. Off. J. Am. Assoc. Cancer Res..

[140] Schmidt M., Volz B., Wittig B., Knäblein J. (2005). MIDGE Vectors and dSLIM Immunomodulators: DNA-based Molecules for Gene Therapeutic Strategies. Modern Biopharmaceuticals.

[141] Köchling J., Prada J., Bahrami M., Stripecke R., Seeger K., Henze G., Wittig B., Schmidt M. (2008). Anti-tumor effect of DNA-based vaccination and dSLIM immunomodulatory molecules in mice with Ph+ acute lymphoblastic leukaemia. Vaccine.

[142] Kobelt D., Aumann J., Schmidt M., Wittig B., Fichtner I., Behrens D., Lemm M., Freundt G., Schlag P.M., Walther W. (2014). Preclinical study on combined chemo- and nonviral gene therapy for sensitization of melanoma using a human TNF-alpha expressing MIDGE DNA vector. Mol. Oncol..

[143] Monjezi R., Miskey C., Gogishvili T., Schleef M., Schmeer M., Einsele H., Ivics Z., Hudecek M. (2016). Enhanced CAR T-cell engineering using non-viral Sleeping Beauty transposition from minicircle vectors. Leukemia.

[144] Pang X., Ma F., Zhang P., Zhong Y., Zhang J., Wang T., Zheng G., Hou X., Zhao J., He C.-Y., Chen Z.-Y. (2016). Treatment of human B-cell lymphomas using minicircle DNA vector expressing anti-CD3/CD20 in a mouse model. Hum. Gene Ther..

[145] Ronald J.A., Chuang H.-Y., Dragulescu-Andrasi A., Hori S.S., Gambhir S.S. (2015). Detecting cancers through tumor-activatable minicircles that lead to a detectable blood biomarker. Proc. Natl. Acad. Sci. USA.

[146] Jia F., Wilson K.D., Sun N., Gupta D.M., Huang M., Li Z., Panetta N.J., Chen Z.Y., Robbins R.C., Kay M.A. (2010). A nonviral minicircle vector for deriving human iPS cells. Nat. Methods.

[147] Daneshvar N., Rasedee A., Shamsabadi F.T., Moeini H., Mehrboud P., Rahman H.S., Boroojerdi M.H., Vellasamy S. (2015). Induction of pluripotency in human umbilical cord mesenchymal stem cells in feeder layer-free condition. Tissue Cell.

[148] Fernandes A.R., Chari D.M. (2016). Part II: Functional delivery of a neurotherapeutic gene to neural stem cells using minicircle DNA and nanoparticles: Translational advantages for regenerative neurology. J. Control. Release Off. J. Control. Release Soc..

[149] Griesenbach U., Geddes D.M., Alton E.W.F.W. (2004). Gene therapy for cystic fibrosis: An example for lung gene therapy. Gene Ther..

[150] Munye M.M., Tagalakis A.D., Barnes J.L., Brown R.E., McAnulty R.J., Howe S.J., Hart S.L. (2016). Minicircle DNA provides enhanced and prolonged transgene expression following airway gene transfer. Sci. Rep..

[151] Driskell R.R., Engelhardt J.F. (2003). Current status of gene therapy for inherited lung diseases. Annu. Rev. Physiol..

[152] Xia E., Munegowda M.A., Cao H., Hu J. (2014). Lung gene therapy—How to capture illumination from the light already present in the tunnel. Genes Dis..

[153] Duncan G.A., Jung J., Hanes J., Suk J.S. (2016). The mucus barrier to inhaled gene therapy. Mol. Ther..

[154] Alton E.W.F.W., Boyd A.C., Cheng S.H., Davies J.C., Davies L.A., Dayan A., Gill D.R., Griesenbach U., Higgins T., Hyde S.C. (2014). Toxicology study assessing efficacy and safety of repeated administration of lipid/DNA complexes to mouse lung. Gene Ther..

[155] Li S., Wu S.-P., Whitmore M., Loeffert E.J., Wang L., Watkins S.C., Pitt B.R., Huang L. (1999). Effect of immune response on gene transfer to the lung via systemic administration of cationic lipidic vectors. Am. J. Physiol.-Lung Cell. Mol. Physiol..

[156] Bazzani R.P., Pringle I.A., Connolly M.M., Davies L.A., Sumner-Jones S.G., Schleef M., Hyde S.C., Gill D.R. (2016). Transgene sequences free of CG dinucleotides lead to high level, long-term expression in the lung independent of plasmid backbone design. Biomaterials.

[157] Matkar P.N., Leong-Poi H., Singh K.K. (2016). Cardiac gene therapy: Are we there yet?. Gene Ther..

[158] Stenler S., Andersson A., Simonson O.E., Lundin K.E., Chen Z.-Y., Kay M.A., Smith C.E., Sylvén C., Blomberg P. (2009). Gene transfer to mouse heart and skeletal muscles using a minicircle expressing human vascular endothelial growth factor. J. Cardiovasc. Pharmacol..

[159] Hou X., Jiao R., Guo X., Wang T., Chen P., Wang D., Chen Y., He C.-Y., Chen Z.-Y. (2016). Construction of minicircle DNA vectors capable of correcting familial hypercholesterolemia phenotype in a LDLR-deficient mouse model. Gene Ther..

[160] Hu S., Huang M., Li Z., Jia F., Ghosh Z., Lijkwan M.A., Fasanaro P., Sun N., Wang X., Martelli F. (2010). MicroRNA-210 as a novel therapy for treatment of ischemic heart disease. Circulation.

[161] Huang M., Nguyen P., Jia F., Hu S., Gong Y., de Almeida P.E., Wang L., Nag D., Kay M.A., Giaccia A.J. (2011). Double knockdown of prolyl hydroxylase and factor-inhibiting hypoxia-inducible factor with nonviral minicircle gene therapy enhances stem cell mobilization and angiogenesis after myocardial infarction. Circulation.

[162] Yoon C.S., Jung H.S., Kwon M.J., Lee S.H., Kim C.W., Kim M.K., Lee M., Park J.H. (2009). Sonoporation of the minicircle-VEGF(165) for wound healing of diabetic mice. Pharm. Res..

[163] Zibert J.R., Wallbrecht K., Schön M., Mir L.M., Jacobsen G.K., Trochon-Joseph V., Bouquet C., Villadsen L.S., Cadossi R., Skov L. (2011). Halting angiogenesis by non-viral somatic gene therapy alleviates psoriasis and murine psoriasiform skin lesions. J. Clin. Invest..

[164] Mohan R.R., Tovey J.C.K., Sharma A., Tandon A. (2012). Gene therapy in the Cornea: 2005–Present. Prog. Retin. Eye Res..

[165] Gene Therapy Clinical Trials Worldwide. http://www.abedia.com/wiley/years.php.

[166] Lijkwan M.A., Hellingman A.A., Bos E.J., van der Bogt K.E.A., Huang M., Kooreman N.G., de Vries M.R., Peters H.A.B., Robbins R.C., Hamming J.F. (2014). Short hairpin RNA gene silencing of prolyl hydroxylase-2 with a minicircle vector improves neovascularization of hindlimb ischemia. Hum. Gene Ther..

[167] Rim Y.A., Yi H., Kim Y., Park N., Jung H., Kim J., Jung S.M., Park S.-H., Ju J.H. (2014). Self in vivo production of a synthetic biological drug CTLA4Ig using a minicircle vector. Sci. Rep..

[168] Yi H., Kim Y., Kim J., Jung H., Rim Y.A., Jung S.M., Park S.-H., Ju J.H. (2014). A new strategy to deliver synthetic protein drugs: Self-reproducible biologics using minicircles. Sci. Rep..

[169] Gaspar V.M., Gonçalves C., de Melo-Diogo D., Costa E.C., Queiroz J.A., Pichon C., Sousa F., Correia I.J. (2014). Poly(2-ethyl-2-oxazoline)-PLA-g-PEI amphiphilic triblock micelles for co-delivery of minicircle DNA and chemotherapeutics. J. Control. Release Off. J. Control. Release Soc..

[170] Gaspar V.M., Baril P., Costa E.C., de Melo-Diogo D., Foucher F., Queiroz J.A., Sousa F., Pichon C., Correia I.J. (2015). Bioreducible poly(2-ethyl-2-oxazoline)-PLA-PEI-SS triblock copolymer micelles for co-delivery of DNA minicircles and doxorubicin. J. Control. Release Off. J. Control. Release Soc..

[171] Gaspar V.M., Moreira A.F., Costa E.C., Queiroz J.A., Sousa F., Pichon C., Correia I.J. (2015). Gas-generating TPGS-PLGA microspheres loaded with nanoparticles (NIMPS) for co-delivery of minicircle DNA and anti-tumoral drugs. Colloids Surf. B Biointerfaces.

[172] Pandey N., Nobles C.L., Zechiedrich L., Maresso A.W., Silberg J.J. (2015). Combining random gene fission and rational gene fusion to discover near-infrared fluorescent protein fragments that report on protein-protein interactions. ACS Synth. Biol..

[173] Mahdavi A., Segall-Shapiro T.H., Kou S., Jindal G.A., Hoff K.G., Liu S., Chitsaz M., Ismagilov R.F., Silberg J.J., Tirrell D.A. (2013). A genetically encoded AND gate for cell-targeted metabolic labeling of proteins. J. Am. Chem. Soc..

[174] Wang Q., Irobalieva R.N., Chiu W., Schmid M.F., Fogg J.M., Zechiedrich L., Pettitt B.M. Influence of DNA sequence and structure of minicircles under torsional stress.

[175] Michnick S.W., Ear P.H., Manderson E.N., Remy I., Stefan E. (2007). Universal strategies in research and drug discovery based on protein-fragment complementation assays. Nat. Rev. Drug Discov..

[176] Ullmann A., Perrin D., Jacob F., Monod J. (1965). Identification, by in vitro complementation and purification, of a peptide fraction of *Escherichia coli* beta-galactosidase. J. Mol. Biol..

